# Adiponectin Knockout Mice Display Cognitive and Synaptic Deficits

**DOI:** 10.3389/fendo.2019.00819

**Published:** 2019-11-22

**Authors:** Jenna Bloemer, Priyanka D. Pinky, Warren D. Smith, Dwipayan Bhattacharya, Alisa Chauhan, Manoj Govindarajulu, Hao Hong, Muralikrishnan Dhanasekaran, Robert Judd, Rajesh H. Amin, Miranda N. Reed, Vishnu Suppiramaniam

**Affiliations:** ^1^Department of Drug Discovery and Development, Harrison School of Pharmacy, Auburn University, Auburn, AL, United States; ^2^Center for Neuroscience, Auburn University, Auburn, AL, United States; ^3^Key Laboratory of Neuropsychiatric Diseases, Department of Pharmacology, China Pharmaceutical University, Nanjing, China; ^4^Department of Anatomy, Physiology and Pharmacology, College of Veterinary Medicine, Auburn University, Auburn, AL, United States

**Keywords:** adiponectin, synaptic plasticity, cognition, AdipoRon, AdipoR1, AdipoR2

## Abstract

Adiponectin is an adipokine that has recently been under investigation for potential neuroprotective effects in various brain disorders including Alzheimer's disease, stroke, and depression. Adiponectin receptors (AdipoR1 and AdipoR2) are found throughout various brain regions, including the hippocampus. However, the role of these receptors in synaptic and cognitive function is not clear. Therefore, the goal of the current study was to evaluate synaptic and cognitive function in the absence of adiponectin. The current study utilized 12-month-old adiponectin knockout (APN-KO) mice and age-matched controls to study cognitive and hippocampal synaptic alterations. We determined that AdipoR1 and AdipoR2 are present in the synaptosome, with AdipoR2 displaying increased presynaptic vs. postsynaptic localization, whereas AdipoR1 was enriched in both the presynaptic and postsynaptic fractions. APN-KO mice displayed cognitive deficits in the novel object recognition (NOR) and Y-maze tests. This was mirrored by deficits in long-term potentiation (LTP) of the hippocampal Schaefer collateral pathway in APN-KO mice. APN-KO mice also displayed a reduction in basal synaptic transmission and an increase in presynaptic release probability. Deficits in LTP were rescued through hippocampal slice incubation with the adiponectin receptor agonist, AdipoRon, indicating that acute alterations in adiponectin receptor signaling influence synaptic function. Along with the deficits in LTP, altered levels of key presynaptic and postsynaptic proteins involved in glutamatergic neurotransmission were observed in APN-KO mice. Taken together, these results indicate that adiponectin is an important regulator of cognition and synaptic function in the hippocampus. Future studies should examine the role of specific adiponectin receptors in synaptic processes.

## Introduction

Adiponectin is an important adipokine known for its role in modulation of metabolic processes including enhancement of insulin sensitivity (1). In recent years, it has become increasingly clear that adiponectin is an essential player in the cross-talk between adipose tissue and brain function. Adiponectin is produced by adipocytes and circulates in various forms including trimers, hexamers, high molecular weight complexes, and a globular form (2). Trimers and hexamers are detectable in cerebral spinal fluid (3), and there is evidence that trimers are the most biologically relevant form in the brain (4). Adiponectin receptors, which include AdipoR1, AdipoR2, and T-cadherin, are expressed in various regions of the brain (5), including the hippocampus, an important area for learning and memory processes (6). Notably, altered adiponectin plasma levels are associated with neurological disorders, including Alzheimer's disease (AD) (7–9) and major depressive disorder (10). Additionally, studies have shown a positive correlation between serum adiponectin levels and cognitive performance in post-menopausal women (11) and middle aged adults at risk for type 2 diabetes (12). While adiponectin signaling in the brain appears to contribute to processes including hippocampal neurogenesis and energy expenditure (13), the role of adiponectin in synaptic and cognitive function is still unknown.

To better understand the role of adiponectin in hippocampal synaptic function, we first identified the synaptic localization of the adiponectin receptors AdipoR1 and AdipoR2. Next, we investigated the effects of adiponectin on cognitive and synaptic functioning by comparing aged adiponectin knockout mice (APN-KO) to age-matched controls using behavioral and electrophysiological techniques. We determined that the adiponectin receptor agonist AdipoRon rescues deficits in synaptic plasticity in the APN-KO mice. Finally, we identified alterations in key synaptic proteins that may underlie the cognitive and synaptic deficits observed in APN-KO mice.

## Materials and Methods

### Animals

Adiponectin knockout (B6;129-*Adipoq*^*tm*1*Chan*^/J) and control (B6129SF2/J) male mice were obtained from The Jackson Laboratory (stock #008195 and #101045, respectively) and aged to 12 months. Mice were group-housed with free access to food and water in a temperature- and humidity-controlled colony room with a 12:12 light/dark cycle (lights on at 6 a.m.). Behavioral experiments were performed starting at ~3 h into the light cycle. All procedures were carried out in accordance with NIH guidelines and approved by Auburn University Animal Care and Use Committee (protocols 2015-2618 and 2019-3510).

### Chemicals

For hippocampal slice experiments, AdipoRon, 2-(4-benzoylphenoxy)-*N*-[1-(phenylmethyl)-4 piperidinyl]acetamide (Cayman Chemical), was dissolved in dimethyl sulfoxide (DMSO) and stored as a stock solution (50 mM). On the day of the experiment, the AdipoRon solution was diluted in artificial cerebral spinal fluid (ACSF) to a final concentration of 15 μM (14). All other chemicals were obtained from Millipore Sigma, unless otherwise specified.

### Synaptic Fractionation

Hippocampal synaptosomes were prepared as previously described (15–17), followed by synaptic fractionation (18). In brief, hippocampi were homogenized in ice-cold modified KREBS (mKREBS) buffer containing (in mM): 118.5 NaCl, 4.7 KCl, 1.18 MgSO_4_, 2.5 CaCl_2_, 1.18 KH_2_PO_4_, 24.9 NaHCO_3_, and 10 dextrose and adjusted to pH 7.4. Homogenate was filtered through three layers of nylon filter (100 μm) and then through a low-protein binding filter (5 μm). The filtered particulate was spun at 1,000 × g for 15 min, and the pellet contained the synaptosomal fraction. Synaptosomes were resuspended in isolation buffer (20 mM HEPES, 100 mM NaCl, 0.5% Triton X-100, pH 7.2), incubated for 15 min, and centrifuged at 12,000 × g for 20 min. The supernatant contained the non-postsynaptic density (non-PSD) fraction. The pellet was incubated in buffer (20 mM HEPES, 0.15 mM NaCl, 1% Triton X-100, 1% deoxycholic acid, 1% SDS, pH 7.5) for 1 h, then centrifuged for 15 min at 10,000 × g. The supernatant contained the PSD fraction, and the pellet was discarded. All buffers were supplemented with Halt Protease and Phosphatase Inhibitor Cocktail (ThermoFischer Scientific), and all steps were carried out at 4°C. Protein concentration was estimated by bicinchoninic acid (BCA) assay (Pierce BCA Protein Assay Kit, ThermoFischer Scientific) and samples were stored at −20°C until use.

### Immunoblotting

Following euthanasia, hippocampal tissue was extracted and homogenized in lysis buffer (Neuronal Protein Extraction Reagent, ThermoFischer Scientific) containing a protease and phosphatase inhibitor cocktail (Halt Protease and Phosphatase Inhibitor Cocktail, ThermoFischer Scientific). Total protein was estimated by BCA assay (Pierce BCA Protein Assay Kit, ThermoFischer Scientific) and stored at −20°C until use. Hippocampal or synaptosomal lysate was mixed thoroughly with 4 × Laemmli buffer (Bio-Rad), heated, and loaded into a handcast 10% polyacrylamide gel. Electrophoresis was performed using the Mini-PROTEAN 3 system (Bio-Rad). The proteins were transferred to PVDF membranes (Amersham Hybond P, GE Healthcare) and blocked with 5% bovine serum albumin (BSA) (VWR) in Tris-buffered saline, 0.1% v/v Tween 20 (TBST) (Bio-Rad) for 2 h. Membranes were washed with TBST and incubated with primary antibody (Table 1) overnight at 4°C. Additional washing steps were performed, followed by incubation in horseradish peroxidase-conjugated secondary antibody (Table 1) for 1 h at room temperature. Primary antibodies and secondary antibodies were diluted in 5% BSA in TSBT and utilized at the dilutions indicated in Table 1. Immunoreactivity was visualized using enhanced chemiluminescence (Amersham ECL Select or Amersham ECL Prime, GE Healthcare) in a FluorChem Q imager system (Proteinsimple). Density of immunoreactivity for each band was measured using AlphaView software (Proteinsimple) and values were normalized to the beta actin levels of corresponding lanes. Membranes were stripped prior to probing for beta actin if the observed molecular weight of the protein of interest was ±10 kDa from the molecular weight of beta actin. Stripping was performed following the protocol provided in the Amersham ECL Western Blotting Detection Reagent booklet. Briefly, the membrane was incubated in stripping buffer containing 100 mM 2-mercaptoethanol, 2% SDS, and 62.5 mM Tris-HCl pH 6.7 at 50°C for 30 min. After washing in TBST, the membrane was blocked for 1 h using 5% BSA in TBST and probed for beta actin as described above. For analysis of phosphorylated proteins, the phosphorylated and total proteins were detected on separate blots and normalized to beta actin prior to comparing relative densities.

**Table 1:** Summary of antibodies and working conditions used in the experiments.

**Antibodies**	**Species**	**Source**	**Catalog #**	**Dilution**
**Primary antibodies**				
AdipoR1	Rabbit	Abcam	ab126611	1:200
AdipoR2	Mouse	Santa Cruz	sc-514045	1:200
SNAP25	Mouse	Santa Cruz	sc-136267	1:2,000
PSD95	Rabbit	Cell Signaling Technology	3409	1:1,000
VGLUT1	Rabbit	Cell Signaling Technology	12331	1:500
GluA1	Rabbit	Cell Signaling Technology	13185	1:750
GluN1	Rabbit	Cell Signaling Technology	5704	1:500
GluN2A	Rabbit	Cell Signaling Technology	4205	1:500
GluN2B	Rabbit	Cell Signaling Technology	4207	1:500
AMPK	Rabbit	Cell Signaling Technology	5832	1:1,000
pAMPK (Thr 172)	Rabbit	Cell Signaling Technology	2531	1:500
GSKβ	Rabbit	Cell Signaling Technology	12456	1:1,000
pGSK3β (Ser 9)	Rabbit	Cell Signaling Technology	5558	1:1,000
CREB	Rabbit	Cell Signaling Technology	4820	1:1,000
pCREB (Ser 133)	Rabbit	Cell Signaling Technology	9198	1:500
Beta actin	Rabbit	Cell Signaling Technology	8457	1:2,000
**Secondary antibodies**				
Anti-mouse IgG	N/A	Santa Cruz	sc-516102	1:2,000
Anti-rabbit IgG	Goat	Cell Signaling Technology	7074	1:5,000

### Novel Object Recognition

To measure recognition memory, novel object recognition (NOR) was performed as previously described (19) with some modifications. NOR was performed in a 40 × 40 cm box made of plexiglass. Prior to the experiment, mice were habituated to an empty box for 10 min on 2 consecutive days. During the familiarization phase, two identical objects (either Object A, green plastic cube measuring 4 cm^3^ or Object B, cylindrical blue cone 5 cm in diameter) were placed in the northeast and northwest quadrants positioned 11 cm from the walls. During the test phase 24 h later, one of the familiar objects was replaced by a novel object (the novel object was Object B if Object A was used as the familiar object, and vice versa). Objects and the arena were cleaned with 70% ethanol between animals to minimize odor cues. During the familiarization and testing phases, mice were placed into the center of the box facing away from the objects and allowed to freely explore for 4 min. Recordings were scored by blinded reviewers. Exploration of the objects was defined as time spent with the snout orientated toward the object at a distance of ≤2 cm of the object. Results were expressed as discrimination index (DI), (T novel – T familiar)/(T familiar + T novel).

### Y-maze Test

Three days after the NOR task, spatial recognition memory utilizing a two-trial Y-maze task was performed as described previously (16). Briefly, the Y-maze apparatus consisted of three arms separated by 120°. Visual cues were placed around the Y-maze. The two trials were separated by a 3-h intertrial interval to assess spatial recognition memory. During the first trial (acquisition), mice were allowed to explore two arms of the maze for 10 min: the starting arm in which they were initially placed, and a second arm referred to as the familiar arm; the third (novel) arm was closed. During the second trial (retention test), mice were placed back in the starting arm and allowed to explore for 6 min with free access to all three arms (the novel arm was open). The arena was cleaned with 70% ethanol between animals to minimize odor cues. Recordings were scored by blinded reviewers, and the total number of entries and time spent in each arm were recorded.

### Hippocampal Slice Preparation

Animals were euthanized with carbon dioxide, and 350-μm thick transverse slices were prepared using a Leica VT1200S Vibratome (Leica Microsystems). Slices were incubated at room temperature in artificial cerebrospinal fluid (ACSF; 124 mM NaCl, 2.5 mM KCl, 1.5 mM MgCl_2_, 2 mM CaCl_2_, 1.25 mM NaH_2_PO_4_, 25 mM NaHCO_3_, 25 mM dextrose, pH 7.4) saturated with 95% O_2_/5% CO_2_ until transfer to the recording chamber. In animals which were behaviorally tested, 1 week elapsed between behavioral testing and electrophysiology experiments.

### Extracellular Field Potential Recording

After 2 h of incubation in either ACSF containing 0.03% DMSO (Vehicle) or ACSF containing 15 μM of AdipoRon and 0.03% DMSO (AdipoRon), slices were transferred into a submerged slice chamber for electrophysiological measurements as previously described (20–23) with continuous ACSF perfusion at 34°C. A bipolar stimulating electrode (MicroProbes) was placed in the Schaffer collateral pathway. An extracellular recording pipette drawn with the PC-10 Dual-Stage Glass Micropipette Puller (Narishige) and filled with ACSF (2–6 MΩ) was placed in the stratum radiatum of CA1 to record field excitatory postsynaptic potentials (fEPSPs). For paired pulse facilitation (PPF), pairs of stimuli were separated by varying intervals. Ratios of fEPSP slopes from the second stimulus (fESPS2) to fEPSP slopes from the first stimulus (fESPS1) were calculated and plotted as a function of interstimulus intervals. Basal synaptic transmission, represented by input-output responses, was determined as the slope of fEPSPs and plotted as a function of fiber volley amplitude. For long-term potentiation (LTP) experiments, stimulus intensity was set at 50% of the amplitude at which initial population spike appeared. LTP was induced after at least 10 min of stable baseline recording using a Theta Burst Stimulation (TBS) protocol (10 bursts of stimuli, each of four pulses at 100 Hz, interburst interval of 200 ms, and 20 s intervals between individual sweeps), and recording was continued for 60 min post-TBS (21, 24, 25). LTP was measured as an average of fEPSP slopes from 50 to 60 min after the end of induction. For the induction data analysis, sweep analysis was computed by normalizing the amplitude of the first fEPSP of sweeps 2–5 with the amplitude of the first fEPSP of the first sweep. The data were recorded online using the WinLTP software (University of Bristol, UK) (26). Standard off-line analyses of the data were conducted using Prism software (GraphPad Prism version 8).

### Statistical Analysis

Statistical analysis was performed using Prism 8 software. Data was assessed by one-way analysis of variance or two-tailed, unpaired and paired *t*-tests, where appropriate. Tukey *post-hoc* comparisons were used to compare groups when analysis of variance indicated significant effects except where expected effects were assessed with planned pairwise comparisons. Results were considered significantly different when *p* < 0.05. All data are presented as means ± SEM.

## Results

### AdipoR1 and AdipoR2 Are Expressed in Hippocampal Synapses

**Figure 1:**
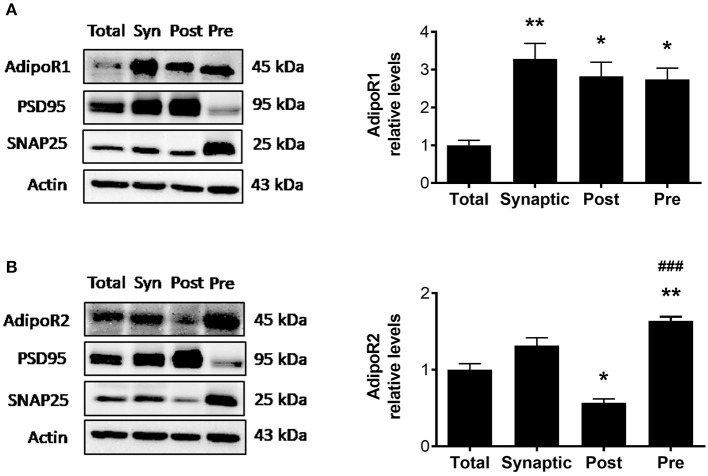
Synaptosomal densities of AdipoR1 and AdipoR2. **(A)** Representative immunoblot showing AdipoR1 immunoreactivity normalized to beta actin in hippocampal fractions. **(B)** Representative immunoblot showing AdipoR2 immunoreactivity normalized to beta actin in hippocampal fractions. Hippocampal lysate was divided into total (Total) and synaptosomal (Syn) fractions. The synaptosome was further fractionated into the PSD (Post) and non-PSD (Pre) fractions. Synaptic fractionation efficiency is represented by immunoreactivity of PSD95 and SNAP25 for Post and Pre, respectively. Twenty five micrograms of protein were loaded per lane. Bars represent mean ± SEM from 3 independent experiments, hippocampi from 2 mice were pooled for each experiment (n = 6 mice); *indicates significant difference vs. Total; ^#^indicates significant difference vs. Post; *p < 0.05, **p < 0.01, ^*###*^*p* < 0.001. Tukey's *post hoc* test was used for multiple comparisons.

Although adiponectin receptors are found throughout the brain including the hippocampus, cortex, hypothalamus, and brainstem (5), whether adiponectin receptors are expressed at the synapse, the functional unit of communication between neurons, is unknown. To determine whether AdipoR1 and AdipoR2 receptors are expressed at hippocampal synapses in wild-type mice, a synaptosomal isolation procedure followed by immunoblotting was used to compare synaptic vs. total hippocampal densities. Synaptic fractionation efficiency was confirmed by immunoblotting for post-synaptic density 95 (PSD95), enriched in the postsynaptic fraction, and synaptosome associated protein 25 (SNAP25), enriched in the presynaptic fraction. In wild-type mice, AdipoR1 levels in various fractions differed such that the AdipoR1 receptor was significantly enriched in the synaptosome compared to the total hippocampal lysate [*F*_(3,8)_ = 9.751, *p* = 0.0048; Figure 1A]. When the synaptosomal fraction was further isolated into the PSD and non-PSD fractions, with the non-PSD fraction containing predominately presynaptic material, there was no significant difference in AdipoR1 levels in postsynaptic vs. presynaptic fraction. However, each was about three times higher compared to the total lysate (Figure 1A), indicating that AdipoR1 receptors may play a role in both presynaptic and postsynaptic processes. The density of AdipoR2 in the synaptosome was similar to the density in total hippocampal lysate. However, AdipoR2 density was significantly higher in the presynaptic fraction compared to the postsynaptic fraction, implying a potential presynaptic role of this receptor [*F*_(3,8)_ = 38.23, *p* < 0.0001; Figure 1B].

### APN-KO Mice Display Deficits in NOR and Y-Maze Tests

**Figure 2:**
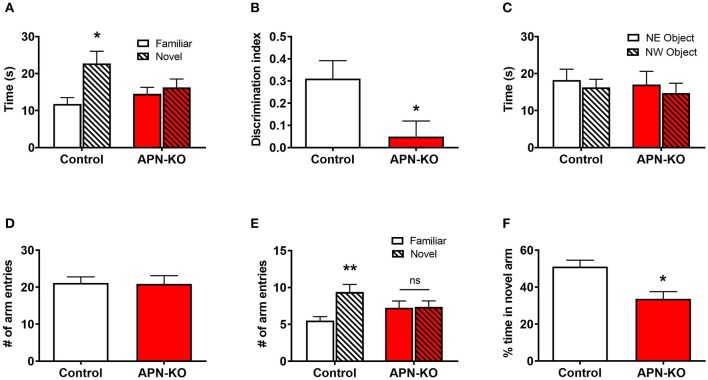
APN-KO mice display deficits in novel object recognition test and in two-trial Y-maze. **(A)** Time spent interacting with the familiar vs. novel object during testing. **(B)** Discrimination index calculated as (T_N_ – T_F_)/(T_N_ + T_F_) where T_N_ is time spent with the novel object and T_F_ is time spent with the familiar object. **(C)** Time spent interacting with the object in the northeast (NE) and northwest (NW) quadrants during familiarization. **(D)** Total number of arm entries during testing **(E)** number of arm entries into the familiar and novel arms during testing **(F)** percent time spent in novel arm during testing. Bars represent mean ± SEM; *p < 0.05, **p < 0.01; *n* = 8 mice per group.

Since adiponectin receptors are expressed in hippocampal synapses, we hypothesized that these receptors may play a role in synaptic processes and that loss of adiponectin may lead to learning and memory deficits. To determine whether cognitive deficits are present in aged APN-KO mice, we performed hippocampal-dependent NOR (19, 27) and Y-maze (16) tests. Using a 24-h separation between the familiarization and test phase for the NOR task to test long term recognition memory (28), control mice were found to spend significantly more time interacting with the novel object compared to the familiar object [*F*_(1,14)_ = 7.622, *p* = 0.0153; Figure 2A], whereas the APN-KO mice showed no preference for the novel object [*F*_(1,14)_ = 0.3167, *p* = 0.5825; Figure 2A]. This was confirmed by comparing the discrimination index, which revealed a significant impairment in the APN-KO mice compared to controls in overall performance in NOR [*F*_(1,14)_ = 5.88, *p* = 0.0294; Figure 2B]. There was no significant difference in exploration time between groups during familiarization [*F*_(1,14)_ = 0.4044, *p* = 0.5825; Figure 2C], indicating that the lack of preference for the novel object in the APN-KO mice was not due to deficits in exploratory behavior.

To determine whether APN-KO mice also display deficits in short-term spatial recognition memory, we utilized a two-trial Y-maze task with an intertrial interval of 3 h. There was no difference in number of total arm entries between groups during testing, indicating a similar level of exploratory behavior [*F*_(1,14)_ = 0.008, *p* = 0.9299; Figure 2D]. While the control mice showed a significant preference for entries into the novel arm [*F*_(1,14)_ = 9.356, *p* = 0.0085; Figure 2E], the APN-KO did not discriminate between the familiar and novel arms [*F*_(1,14)_ = 0.0092, *p* = 0.9251; Figure 2E]. Likewise, the control mice spent a significantly higher percentage of time in the novel arm compared to the APN-KO mice [*F*_(1,14)_ = 11.11, *p* = 0.0049; Figure 2F]. Taken together, this data indicates cognitive impairment in the APN-KO mice.

### Deficits in Basal Synaptic Transmission in APN-KO Mice Are Rescued by Slice Incubation With AdipoRon

**Figure 3:**
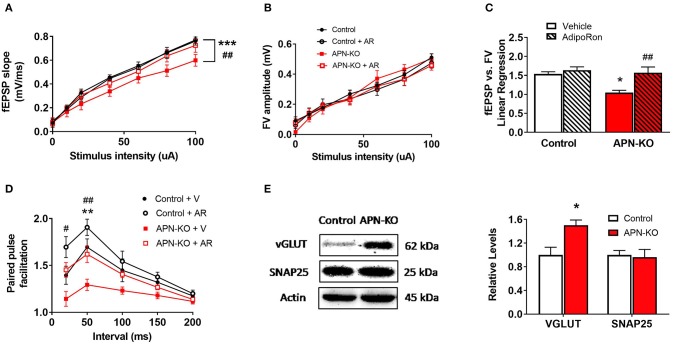
APN-KO mice display alterations in basal synaptic transmission and paired pulse facilitation which are rescued through incubation with AdipoRon. For **(A–D)**, hippocampal slices were prepared from Control and APN-KO mice and incubated for 2-h in either ACSF containing 0.03% DMSO [Vehicle (V)] or ACSF containing 15 μM of AdipoRon and 0.03% DMSO [AdipoRon (AR)] prior to recording. **(A)** Input-output curve of fEPSP slope measured at increasing stimulus intensities. **(B)** Input-output curve of FV amplitude measured at increasing stimulus intensities. **(C)** Slope of the linear regression line of best fit from plotting fEPSP slope vs. FV amplitude. **(D)** Paired-pulse facilitation expressed as the change in ratio of the second stimulus fEPSP to the first stimulus fEPSP slope plotted as a function of interstimulus interval. **(E)** Representative immunoblot showing vGLUT1 and SNAP25 relative densities normalized to beta actin in total hippocampal lysate. Forty micrograms of protein were loaded per lane. Symbols/bars represent mean ± SEM; *indicates significant difference between APN-KO and Control, ^#^indicates significant difference between APN-KO and APN-KO + AR; */^#^*p* < 0.05, **/^*##*^*p* < 0.01, ***p < 0.001; for **(A–D)**, *n* = 5–6 slices from 4 mice per group; for **(E)**, *n* = 4 mice per group; for **(A–C)**, Tukey's *post hoc* test was used for multiple comparisons; for **(D)**, planned pairwise comparisons were performed for individual data point analysis for Control vs. APN-KO, APN-KO vs. APN-KO + AR, and Control vs. APN-KO + AR.

To determine whether cognitive impairment in the APN-KO mice is linked to alterations in basal synaptic transmission, hippocampal slices were used to measure the fEPSP responses at increasing stimulus intensities. We observed an alteration in fEPSPs over a range of stimulus intensities among groups [*F*_(3,18)_ = 13.73, *p* < 0.0001; Figure 3A]. fEPSP slopes were reduced in APN-KO mice compared to control (*p* = 0.0001), implying deficits in baseline glutamatergic synaptic transmission. These deficits were rescued by slice incubation with AdipoRon (*p* = 0.0032). To determine whether the deficits in basal synaptic transmission in APN-KO mice are due to alterations in presynaptic axon recruitment, stimulus intensity vs. fiber volley (FV) amplitude was compared and found to be similar among groups [*F*_(3,18)_ = 0.8927, *p* = 0.4639; Figure 3B], suggesting that the changes in basal synaptic transmission are not due to changes in axon recruitment. Further supporting this, when controlling for FV amplitude, the fEPSPs remained significantly lower in the APN-KO mice, an effect which was rescued by AdipoRon exposure [*F*_(3,16)_ = 7.756, *p* = 0.002; Figure 3C]. Taken together, this suggests that the reduction in basal synaptic transmission is not due to alterations in presynaptic axon recruitment.

### APN-KO Mice Display Alterations in Presynaptic Release Probability

To determine whether alterations in presynaptic release probability could account for the changes in basal synaptic transmission, we evaluated PPF, a type of short-term plasticity that depends on residual calcium build-up in the presynaptic terminal. Surprisingly, we found a decrease in PPF in the APN-KO mice [*F*_(3,12)_ = 11.36, *p* = 0.0008; Figure 3D], which indicates an increase in presynaptic release probability. Interestingly, AdipoRon incubation significantly increased PPF in APN-KO slices (*p* = 0.0093 for the 50 ms interpulse interval), and there was a trend toward an increase in PPF in control slices following AdipoRon incubation (*p* = 0.087 for the 50 ms interpulse interval). To investigate whether changes in presynaptic proteins involved in glutamate release may account for the alteration in PPF in APN-KO mice, the levels of vesicular glutamate transporter 1 (VGLUT1), which is responsible for uptake of glutamate into synaptic vesicles, and SNAP25, which plays an essential role in release of neurotransmitters, were evaluated in whole hippocampal extracts. While SNAP25 density did not differ between groups [*T*_(6)_ = 0.2513, *p* = 0.81; Figure 3E], VGLUT1 density was significantly increased in APN-KO mice [*T*_(6)_ = 3.176, *p* = 0.0192; Figure 3E], suggesting that an increase in vesicular glutamate storage may underlie the increase in glutamate release observed in APN-KO mice.

### Deficits in LTP in APN-KO Mice Are Rescued Through Slice Incubation With AdipoRon

**Figure 4:**
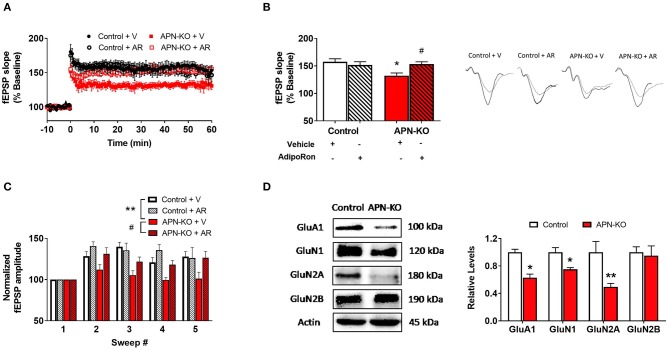
Deficits in LTP in APN-KO mice are associated with reduced levels of glutamatergic receptor subunits. For **(A–C)**, hippocampal slices were prepared from Control and APN-KO mice and incubated for 2-h in either ACSF containing 0.03% DMSO [Vehicle (V)] or ACSF containing 15 μM of AdipoRon and 0.03% DMSO [AdipoRon (AR)] prior to recording. **(A)** LTP graph represents fEPSP slope before and after induction by TBS. **(B)** LTP bar graph shows fEPSPs recorded during the time period 50–60 min following TBS induction normalized to baseline levels, and traces before and after LTP induction are shown. **(C)** Sweep analysis computed by normalizing the amplitude of the first fEPSP of sweeps 2–5 with the amplitude of the first fEPSP of sweep 1 during LTP induction. **(D)** Representative immunoblot showing GluA1, GluN1, GluN2A, and GluN2B relative levels normalized to beta-actin in total hippocampal lysate. Forty micrograms of protein were loaded per lane. Bars represent mean ± SEM; *indicates significant difference between APN-KO and Control, ^#^indicates significant difference between APN-KO and APN-KO + AR; */^#^*p* < 0.05, **p < 0.01; for **(A–C)**, *n* = 5–6 slices from 4 mice per group; for **(D)**, *n* = 3–4 mice per group; Tukey's *post hoc* test was used for multiple comparisons.

We next examined whether cognitive impairments in APN-KO mice are associated with alterations in synaptic plasticity by measuring LTP, which has been termed the cellular correlate of learning and memory (29). Using hippocampal slices, we determined that there was a significant difference among groups when APN-KO and control slices were exposed to AdipoRon or vehicle [*F*_(3,15)_ = 4.899, *p* = 0.0144; Figures 4A,B]. APN-KO mice displayed deficits in LTP induced by TBS in the Schaeffer collateral pathway compared to control mice (*p* = 0.0145). These deficits in APN-KO mice were rescued by the adiponectin receptor agonist, AdipoRon (*p* = 0.0445), suggesting that adiponectin receptor signaling may directly influence glutamatergic synaptic processes in the hippocampus.

A reduction in LTP could indicate alterations in the strength of the signaling during LTP induction (29). To assess for alterations during LTP induction, we evaluated fEPSP amplitude during TBS and found a significant difference among groups [*F*_(3,12)_ = 9.989, *p* = 0.0014; Figure 4C] (21). When the first fEPSP from each sweep was normalized to the first fEPSP of the first sweep, we found a reduction in the fEPSP amplitude in the APN-KO mice compared to controls (*p* = 0.0057), indicating that alterations in LTP may be in part due to reduced synaptic activation during LTP induction. Incubation with AdipoRon incubation restored the fEPSP amplitude during induction (*p* = 0.024).

### Reduced Levels of Glutamatergic Receptor Subunits in APN-KO Mice

Since altered levels of α-amino-3-hydroxy-5-methyl-4-isoxazolepropionic acid receptor (AMPAR) or N-methyl-D-aspartate receptor (NMDAR) subunits can lead to changes in the induction and maintenance of LTP (29), we next evaluated the densities of key subunits via Western Blot in whole hippocampal extracts. The AMPAR subunit GluA1 was reduced in APN-KO mice [*T*_(4)_ = 5.526, *p* = 0.0052; Figure 4D], which may contribute to deficits in LTP along with deficits in basal synaptic transmission. Additionally, there was a reduction in the levels of GluN1 [*T*_(6)_ = 3.465, *p* = 0.0134; Figure 4D] and GluN2A [*T*_(6)_ = 3.046, *p* = 0.0226; Figure 4D], NMDAR subunits found in the postsynaptic zone (30, 31). No change in the density of GluN2B was observed in APN-KO mice [*T*_(6)_ = 0.3035, *p* = 0.7717; Figure 4D]. Taken together, our data indicates that reduced levels of glutamatergic receptor subunits may account for the alteration in synaptic plasticity in APN-KO mice.

### Altered Phosphorylation of Downstream Signaling Molecules in APN-KO Mice

**Figure 5:**
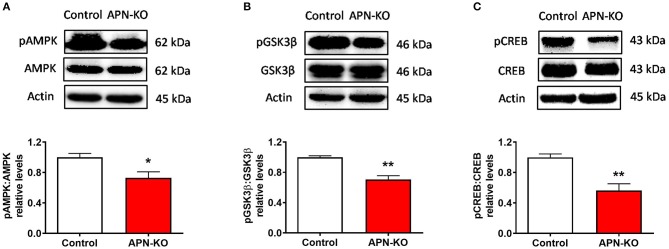
Altered phosphorylation of downstream signaling molecules in APN-KO mice. Representative immunoblots showing **(A)** pAMPK/AMPK relative densities **(B)** pGSK3β/GSK3β relative densities and **(C)** pCREB/CREB relative densities, in total hippocampal lysate. Forty micrograms of protein were loaded per lane. Bars represent mean ± SEM; *p < 0.05, **p < 0.01; *n* = 4 mice per group.

To identify potential mechanisms by which a reduction in adiponectin receptor signaling might result in synaptic alterations, specifically alterations in LTP, we evaluated the densities of key downstream signaling molecules for AdipoR1 in whole hippocampal extracts. Phosphorylation of AMP-activated protein kinase (AMPK), a major downstream signaling molecule for AdipoR1 (32) and an important regulator of LTP (33), was significantly reduced in APN-KO mice [*T*_(6)_ = 2.855, *p* = 0.029; Figure 5A]. AMPK leads to inactivation of glycogen synthase kinase 3 β (GSK3β) through phosphorylation of Ser9, which enhances LTP (34). In APN-KO mice, the pGSK3β/GSK3β ratio was reduced [*T*_(6)_ = 5.45, *p* = 0.0016; Figure 5B], indicating increased activation of GSK3β. cAMP response element-binding protein (CREB) activity is reduced by GSK3β (35), and CREB is important in promoting the expression of glutamatergic receptor subunits (36, 37). In APN-KO mice, the pCREB/CREB ratio was reduced [*T*_(6)_ = 4.39, *p* = 0.0046; Figure 5C], indicating lower activity of CREB. Thus, the deficits in LTP in APN-KO mice may be related to a reduction in AdipoR1-mediated signaling.

## Discussion

The key findings of the current study are that adiponectin receptors are expressed in hippocampal synapses, and adiponectin receptor signaling influences synaptic processes. Densities of the adiponectin receptors in the hippocampal synaptosome indicate a potential role for AdipoR1 in both presynaptic and postsynaptic compartments and a role for AdipoR2 in the presynaptic compartment. This led us to hypothesize that the adiponectin receptors may play a direct role in synaptic function and ultimately learning and memory. In APN-KO mice, signaling through both AdipoR1 and AdipoR2 are presumably reduced. In the current study, reduced adiponectin receptor signaling led to deficits in NOR and Y-maze tasks. In accordance with this, 18-month-old APN-KO display impaired performance in Morris Water Maze (38), a hippocampal-dependent memory task. In an AD model, treatment with the adiponectin receptor agonist, osmotin, improves performance in the Y-maze spontaneous alternations test (39). Thus, our findings are in line with prior studies which indicate a positive role for adiponectin receptor signaling in learning and memory. Interestingly, AdipoR1 and AdipoR2 may play distinct roles in learning and memory processes. Knockdown of AdipoR1 leads to impaired spatial memory and development of an AD-like pathology (40). In contrast, AdipoR2, but not AdipoR1, knockout mice show impaired contextual fear extinction (14), implying a role for AdipoR2 in specific learning paradigms. The differential levels of adiponectin receptors in various compartments observed in the current study may relate to different roles of these receptors in learning and memory processes. Future studies should determine whether treatment with AdipoRon restores cognitive deficits in APN-KO mice, as well as whether APN-KO mice exhibit alterations in measures of anxiety or locomotion that may confound interpretations of memory assessments.

The deficits in basal synaptic transmission observed in the current study indicate deficits in glutamatergic signaling in the hippocampus in APN-KO mice. The relationship between the slope of the fEPSP and the FV amplitude is the ideal indicator of basal synaptic transmission, because it represents the postsynaptic response after controlling for the activation of presynaptic fibers (41). Various factors may contribute to a reduction in basal synaptic transmission in glutamatergic synapses including reduction in the release of glutamate, altered glutamate uptake, or decreased levels of glutamatergic receptors, specifically AMPARs (41). In the current study, the reduction in basal synaptic transmission may be related to a reduction in the density of the AMPAR subunit, GluA1. One possible explanation for the reduction in GluA1 density could be due to the increase in presynaptic release probability observed in APN-KO mice, as GluA1 may be downregulated in response to enhanced glutamate exposure (42). Alternatively, an absence of AdipoR1 signaling could lead to reduced activation of CREB and thus reduced expression of GluA1 (37). A recent study showed that adiponectin receptor signaling may directly influence the trafficking of GluA1-containing AMPARs and increase surface density in an AD model (43). Future studies should determine whether treatment with AdipoRon restores levels of GluA1 in APN-KO mice.

In the current study, the increase in presynaptic release probability in APN-KO mice was mirrored by an increase in VGLUT1 density. VGLUTs are responsible for uptake of glutamate into synaptic vesicles in presynaptic terminals, and VGLUT1 is the primary isoform found in the hippocampus (44). The level of VGLUT is related to the number and glutamate fill state of presynaptic vesicles, and VGLUT density is correlated with release probability in hippocampal neurons (45, 46). In our study, no change was observed in the level of SNAP25, which facilitates synaptic transmission by mediating synaptic vesicle exocytosis (47). Thus, the increase in presynaptic release probability observed in the current study may be due to an increase in vesicular glutamate packaging or the number of synaptic vesicles. Future studies should determine whether treatment with AdipoRon restores levels of VGLUT in APN-KO mice.

Interestingly, there was a trend toward reduction in presynaptic release probability after AdipoRon incubation in control slices. This indicates that further activation of adiponectin receptors may alter presynaptic parameters even when basal levels of adiponectin are present. The mechanism for presynaptic alteration is unclear, however, enhancement of macroautophagy is one potential mechanism by which adiponectin receptor signaling could influence the presynaptic compartment. Macroautophagy is a process that involves encapsulation of cellular components into an autophagosome, followed by transportation of the components to the lysosome for degradation and recycling (48). Enhancement of autophagy in presynaptic neuronal terminals leads to a reduction in neurotransmitter release due to a reduction in the number of presynaptic vesicles (49). One possibility is that the increase in VGLUT in the current study represents an increase in the availability of presynaptic vesicles in APN-KO mice. In peripheral tissues, adiponectin receptor signaling enhances autophagy through both AdipoR1 and AdipoR2 mediated mechanisms (50–52). Our data suggests that both AdipoR1 and AdipoR2 are enriched in the presynaptic compartment, so AdipoRon may alter presynaptic parameters by AdipoR1- and AdipoR2- mediated enhancement of autophagy. Whether adiponectin receptor signaling affects neuronal autophagy is unclear and should be determined experimentally. Alternatively, alterations in PPF in response to AdipoRon incubation may be related to effects of adiponectin receptor activation on GABAergic neurotransmission. Activation of GABA receptors contributes to PPF (53, 54), and an effect of adiponectin receptor signaling on GABAergic neurons cannot be ruled out.

In addition to altering basal synaptic transmission and presynaptic release probability, adiponectin receptor signaling also influences synaptic plasticity. The reduction of LTP in APN-KO mice indicates an impairment in synaptic strengthening, which also occurs in neurodegenerative disorders such as AD (55). Importantly, incubation with an adiponectin receptor agonist restored LTP, indicating that the deficits in APN-KO mice are reversed with acute restoration in adiponectin receptor signaling. Likewise, adiponectin incubation restored deficits in LTP in an AD model (43), which was associated with increased surface densities of glutamatergic receptor subunits. Thus, acute adiponectin receptor signaling may improve LTP deficits. However, AdipoRon did not increase LTP in control mice in our study, indicating that there may be a ceiling effect related to the ability of adiponectin receptor signaling to enhance synaptic plasticity.

The reduction in LTP in APN-KO mice in the current study may be related to reduction in the levels of glutamatergic receptor subunits. The induction of NMDAR-dependent LTP involves concurrent activation of the glutamatergic receptors AMPA and NMDA (56, 57). Specifically, during LTP induction, activation of AMPARs leads to removal of Mg^2+^ blockage from NMDARs to allow Ca^2+^ influx and subsequent downstream signaling (29). This downstream signaling pathway induces alterations in AMPAR expression and trafficking which are in part responsible for maintenance of LTP (29). Thus, in the current study, reduced levels of AMPAR and NMDAR subunits may be responsible for the impairments in LTP induction and maintenance. We observed a moderate reduction in LTP in APN-KO slices compared to control slices (~43% reduction). In a recent study, removal of GluA1 in hippocampal CA1 neurons led to a moderate reduction in synaptic transmission and complete loss of LTP (58). In the current study, AdipoKO hippocampal lysate showed a reduction in density of various glutamatergic receptor subunits including GluA1 (~37% decrease), GluN1 (~25% decrease), and GluN2A (~50% decrease). Thus, it is possible that the reduction in glutamatergic subunits observed in the current study, especially GluA1, may relate to the reduction in LTP and reduction in basal synaptic transmission. It is not clear whether AdipoRon incubation increases the total levels of glutamatergic receptor subunits, however, a prior study found that adiponectin incubation increases surface expression of AMPAR and NMDAR subunits in brain slices from an AD mouse model (43). In contrast, another study found a reduction in the AMPAR:NMDAR ratio following incubation of hippocampal slices in a higher dose of AdipoRon compared to the current study (59). Thus, adiponectin receptor activation may exhibit differential effects on glutamatergic receptors under different conditions.

The alterations in glutamatergic receptor subunits in APN-KO mice may be due to the reduced activation of downstream signaling molecules of adiponectin receptors. In the current study, we focused our efforts on downstream signaling of AdipoR1, because our synaptic fractionation indicated that AdipoR1 may be enriched in the synapse. AMPK, a downstream signaling molecule of AdipoR1 and an important energy sensing molecule, is highly expressed in the brain (60). Pharmacological inhibition of AMPK impairs LTP (33, 61), however, there is also evidence that overactivation of AMPK impairs LTP, implying that a fine balance is required (62). AMPK inhibits GSK3β, another important regulator of synaptic plasticity. In the current study, we observed a reduction in AMPK phosphorylation, indicating reduced activation, and a reduction of GSK3β phosphorylation, which indicates increased activation, in APN-KO mice. This AMPK-GSK3β signaling pathway has been proposed to lead to AD-like cognitive deficits in states of adiponectin deficiency (38). We also observed a reduction in pCREB in APN-KO mice, which may be a result of increased GSK3β activity (35). Importantly, CREB activation leads to upregulation of glutamatergic receptor subunits, so the reduction in pCREB could account for the reduced levels of glutamatergic receptor subunits in APN-KO mice. Future studies should determine whether treatment with AdipoRon restores phosphorylation levels of these signaling proteins in APN-KO mice. Although the findings from the current study suggest a synaptic role of adiponectin, it is important to note that an influence of adiponectin receptor signaling on glial cells and/or non-synaptic neuronal locations cannot be ruled out. Based on the current study, we hypothesize that adiponectin receptor activation influences hippocampal processes via influencing glutamatergic synapses, however, a direct effect of adiponectin receptor activation on glutamatergic synapses must be confirmed in future mechanistic studies.

In conclusion, our results indicate that adiponectin receptors are present in hippocampal synapses where they may influence synaptic processes and ultimately cognitive function. Absence of adiponectin leads to cognitive deficits, reduced basal synaptic transmission, increased presynaptic release probability, impaired synaptic plasticity, and altered glutamatergic receptor levels. The role of specific adiponectin receptors in synaptic processes warrants further investigation. Additionally, our study supports investigation into the use of adiponectin receptor agonists in conditions associated with synaptic dysfunction and reduced adiponectin receptor signaling.

## Data Availability Statement

The datasets generated for this study are available on request to the corresponding authors.

## Ethics Statement

The animal study was reviewed and approved by Auburn University Animal Care and Use Committee.

## Author Contributions

JB, RJ, RA, MR, and VS conceived and designed the experiments. JB, PP, WS, DB, MG, HH, MD, and AC performed the experiments and/or analyzed the data. JB wrote the manuscript with support from MR and VS. All authors provided input in data interpretation and manuscript review.

### Conflict of Interest

The authors declare that the research was conducted in the absence of any commercial or financial relationships that could be construed as a potential conflict of interest.
